# A Combined Experimental, Theoretical, and Simulation Approach to the Effects of GNPs and MWCNTs on Joule Heating Behavior of 3D Printed PVDF Nanocomposites

**DOI:** 10.3390/polym17212835

**Published:** 2025-10-24

**Authors:** Giovanni Spinelli, Rosella Guarini, Rumiana Kotsilkova, Evgeni Ivanov, Vladimir Georgiev

**Affiliations:** 1 Faculty of Transport Sciences and Technologies, University of Study “Giustino Fortunato”, Via Raffaele Delcogliano 12, 82100 Benevento, Italy; 2 Open Laboratory on Experimental Micro and Nano Mechanics, Institute of Mechanics, Bulgarian Academy of Sciences, Acad. G. Bonchev Str., Block 4, 1113 Sofia, Bulgaria; rguarini@imbm.bas.bg (R.G.); ivanov_evgeni@imbm.bas.bg (E.I.); vgeorgiev@imbm.bas.bg (V.G.); 3 Department of Industrial Engineering, University of Salerno, Via Giovanni Paolo II, 84084 Fisciano, Italy; 4 National Centre of Excellence Mechatronics and Clean Technologies, 8 bul. Kliment Ohridski, 1756 Sofia, Bulgaria

**Keywords:** electric heating, thermal and electrical conductivity, simulation study, de-icing

## Abstract

The thermal behavior of 3D-printed polyvinylidene fluoride (PVDF)-based composites enhanced with carbon nanotubes (CNTs), graphene nanoplatelets (GNPs), and their hybrid formulations was investigated under Joule heating at applied voltages of 2, 3, and 4 V. The influence of filler type and weight fraction on both electrical and thermal conductivity was systematically assessed using a Design of Experiments (DoE) approach. Response Surface Methodology (RSM) was employed to derive an analytical relationship linking conductivity values to filler loading, revealing clear trends and interaction effects. Among all tested formulations, the composite containing 6 wt% of GNPs exhibited the highest performance in terms of thermal response and electrical conductivity, reaching a steady-state temperature of 88.1 °C under an applied voltage of just 4 V. This optimal formulation was further analyzed through multiphysics simulations, validated against experimental data and theoretical predictions, to evaluate its effectiveness for potential practical applications—particularly in de-icing systems leveraging Joule heating. The integrated experimental–theoretical–numerical workflow proposed herein offers a robust strategy for guiding the development and optimization of next-generation polymer nanocomposites for thermal management technologies.

## 1. Introduction

Conductive polymer composites (CPCs) represent a compelling class of multifunctional materials that integrate the flexibility, lightness, and processability of polymers with the superior electrical and thermal properties of conductive nanofillers. In recent years, growing demand for adaptable and lightweight electrothermal systems has stimulated significant research into CPCs for Joule heating applications, where electrical energy is transformed into heat via resistive dissipation [1,2]. This phenomenon forms the core mechanism behind various technologies, including self-regulating heaters, wearable thermal devices, de-icing layers, anti-fogging systems, and thermotherapeutic patches [3,4,5,6].

Historically, one of the most extensively investigated matrices for these composites has been epoxy resin, prized for its mechanical strength, chemical resistance, and thermal stability. When reinforced with carbon-based nanofillers such as carbon nanotubes (CNTs), graphene nanoplatelets (GNPs), and carbon black (CB), epoxy systems have demonstrated impressive gains in electrical conductivity, often reaching percolation thresholds at filler concentrations as low as 0.1–1 wt% depending on dispersion quality and processing methods [7,8,9]. In their seminal work, Jang et al. [10] demonstrated that CNT/polymer composites could be used as an efficient de-icing coating, offering rapid thermal response and high cycling stability. Similarly, Sanchez-Romate et al. [11] developed multifunctional GNP/epoxy-based coatings, demonstrating their strain-sensing ability as well as their Joule heating performance for anti-icing and de-icing applications. Moreover, hybrid systems containing both CNTs and GNPs in epoxy matrices have been investigated for their synergistic effects. The combination of 1D and 2D nanostructures was found to significantly improve percolation behavior and Joule heating efficiency compared to single-filler systems, as shown by Prolongo et al. [12] and Al-Saleh [13]. Despite these promising results, epoxy-based systems inherently suffer from brittleness and limited flexibility, which restrict their applicability in deformable or wearable technologies. In addition, their irreversible curing process and relatively high processing temperatures impose design constraints that are incompatible with soft and conformable electronic systems. As an alternative, thermoplastic polymers, and, in particular, poly(vinylidene fluoride) (PVDF), have emerged as attractive matrices for electrothermal applications. PVDF offers a unique balance of thermal stability, mechanical flexibility, and electrochemical durability, coupled with a high dielectric constant and inherent piezoelectric and pyroelectric properties [14]. Unlike thermosetting epoxies, PVDF is processable by melt-based techniques and retains significant ductility even when loaded with nanofillers, enabling the fabrication of bendable, stretchable, or even woven heating elements. A key challenge in developing conductive PVDF nanocomposites lies in achieving effective dispersion and network formation of the conductive phase within a relatively non-conductive and chemically inert matrix. Here again, carbon-based nanofillers such as multi-walled carbon nanotubes (MWCNTs) and graphene nanoplatelets (GNPs) have proven instrumental. MWCNTs, with their high aspect ratio and excellent intrinsic conductivity, enable the formation of long-range percolated networks even at low loading levels. However, their hydrophobic surface and strong van der Waals interactions often lead to aggregation, especially in polar matrices like PVDF [15,16]. In contrast, GNPs provide an extended surface area and a two-dimensional morphology conducive to forming layered, interconnected conductive pathways. Their stronger compatibility with PVDF, stemming from the matrix’s dipolar nature and high surface energy, promotes more uniform dispersion and improved interfacial contact [17]. These interactions are crucial not only for electrical performance but also for thermal conductivity and mechanical stability. Recent literature has increasingly focused on hybrid filler strategies, wherein MWCNTs and GNPs are co-dispersed within the PVDF matrix in tailored proportions to exploit their complementary geometries and percolation behaviors. In such systems, MWCNTs serve as bridges across GNP “islands,” filling interstitial gaps and facilitating charge transport across disconnected graphene domains. This can result in more robust and redundant conductive networks, enhancing both the electrical conductivity and the uniformity of Joule heating [18,19,20]. Moreover, the influence of nanofiller synergy extends beyond simple conductivity enhancement. The electrothermal response—including heating rate, temperature uniformity, and power efficiency—is highly sensitive to the morphology and spatial distribution of the conductive domains. For instance, Lu et al. [21] reported biodegradable PCL-based nanocomposites reinforced with hybrid GNP/CNT fillers, achieving simultaneous enhancement of Joule heating efficiency and PTC intensity compared to single-filler systems. Similarly, hybrid nanofiller systems have demonstrated improved thermal cycling stability and reduced hotspot formation compared to their single-filler counterparts [22]. Despite these advances, a systematic understanding of how nanofiller type, ratio, and dispersion quality affect the Joule heating performance of PVDF-based composites remains incomplete. In particular, few studies have directly correlated the microstructural characteristics observed via scanning electron microscopy (SEM) with the macro-scale thermal behavior of hybrid nanocomposites under electrical stimulation.

In more recent years, additive manufacturing (AM) has emerged as a promising strategy for fabricating functional polymer-based nanocomposites, particularly for applications involving Joule heating. Among various thermoplastics, poly(vinylidene fluoride) (PVDF) has gained considerable attention due to its favorable combination of electroactive behavior, chemical resistance, and thermal stability. Recent studies have demonstrated that incorporating carbon-based nanofillers such as graphene nanoplatelets (GNPs), carbon nanotubes (CNTs), and carbon black (CB) into PVDF matrices significantly improves electrical conductivity and heating performance. When processed via 3D printing, especially fused filament fabrication (FFF), the orientation and distribution of fillers can be finely controlled, enhancing the percolation networks essential for efficient heat generation. For instance, in our previous study [23], the authors successfully printed PVDF composites with graphene nanoplatelets, showing that heating efficiency is strongly influenced by filament orientation and print parameters. Kausar et al. [24] reviewed 3D and 4D printing of polymer/graphene composites, emphasizing the potential of anisotropic filler alignment in enhancing electrical conductivity for thermal applications.

Elder et al. [25] discussed nanomaterial patterning via 3D printing and how print direction, infill density, and layer height affect the formation of conductive paths—effects especially relevant for PVDF systems. In parallel, Tirado-Garcia et al. [26] observed directional dependence of electrical and thermal properties in high CB-loaded PLA composites, suggesting similar trends in PVDF nanocomposites. Although PVDF is more challenging to print than common polymers, the works by Guvendiren et al. [27] and Ribeiro et al. [28] demonstrated advances in printable formulations and strategies to fabricate complex, electroactive PVDF-based structures. These developments pave the way for multifunctional devices with integrated heating, sensing, and actuation capabilities. In summary, combining PVDF with carbon-based fillers and leveraging the design flexibility of 3D printing enables the creation of advanced, lightweight, and customizable Joule heating devices—ideal for applications in wearable electronics, de-icing systems, and thermal actuators. In this work, we present a systematic and multidisciplinary investigation of PVDF-based nanocomposites reinforced with multi-walled carbon nanotubes (MWCNTs), graphene nanoplatelets (GNPs), and their hybrid combinations, with a particular focus on their electrical conductivity and electrothermal performance under Joule heating.

While many works have focused on either the experimental characterization of electrical and thermal properties or on numerical simulations, our study uniquely combines an integrated experimental–theoretical–numerical workflow that provides a quantitative and predictive framework for material optimization. The approach integrates: (i) a systematic Design of Experiments (DoE) to rigorously evaluate the effects of filler morphology and concentration on electrical and thermal conductivities; (ii) an analytical model based on Response Surface Methodology (RSM) that establishes quantitative correlations between filler loading and resulting electrothermal performance, offering a generalizable tool for predictive, design-driven material development; and (iii) validated multiphysics simulations of the optimized formulation to verify theoretical assumptions, predict transient and steady-state temperature distributions, and explore device-level Joule-heating behavior under realistic boundary conditions, including conduction and convection phenomena.

Previous studies on CNT- or GNP-filled PVDF composites have typically focused on isolated aspects and did not establish quantitative correlations between filler composition, electrical response, and electrothermal behavior. This closed-loop integration enables cross-validation among experimental data, analytical predictions, and numerical simulations, providing robust, predictive insight into both macroscopic performance and the microscale mechanisms governing heat generation and transport—an aspect not simultaneously addressed in prior CNT/GNP–PVDF works. The formulation containing 6 wt% GNPs emerges as the most effective configuration, combining high electrical conductivity with a strong and stable thermal response. Microstructural characterization via SEM, coupled with electrical measurements and controlled Joule-heating experiments, reveals the formation of interconnected conductive networks, localized Joule-heating hotspots, and efficient thermal transport pathways within the polymer matrix. The multiphysics simulations further quantify spatial and temporal temperature distributions, enabling prediction of de-icing performance and operational limits under variable applied voltages, confirming the real-world applicability and scalability of the proposed workflow. Altogether, this study significantly advances current understanding of polymer-based electrothermal composites by elucidating the interplay between filler microstructure, percolation-driven conduction, and electrothermal performance, and establishes a reproducible, quantitatively validated, and predictive pathway for developing next-generation, scalable heating elements for smart textiles, wearable electronics, and thermal management systems.

## 2. Materials and Methods

### 2.1. Materials

The polyvinylidene fluoride (PVDF) Kynar^®^ 721 (powder form) by Arkema (Philadelphia, PA, USA), homopolymer grade, with MW (molecular weight) 450,000 g/mol, MFR (melt flow rate) 15 g/10 min (230 °C, 3.8 Kg), melting point 168 °C, and glass transition temperature −40 °C was used. The selected nanofillers were graphene nanoplatelets (SE1233-GNPs), supplied by The Sixth Element (Changzhou, China), and multi-walled carbon nanotubes (MWCNTs), from Nanocyl NC7000 (Sambreville, Belgium). Some of the main characteristics reported by the producers are shown in Table 1.

### 2.2. Preparation of Nanocomposites and Test Samples

The PVDF-based nanocomposites incorporating GNP and MWCNT and their hybrid combinations, GNP/MWCNT, were fabricated by the melt extrusion technique. The polymer and the fillers were dried at 80 °C for 4 h in a vacuum oven, then the PVDF powder was wrapped with the appropriate amount of GNP or MWCNT in a ball mill for 2 h at a speed of 70 rpm. Then the wrapped powders were extruded in a twin-screw extruder, Teach-Line ZK25T (COLLIN Lab & Pilot Solutions GmbH, Maitenbeth, Germany), at temperatures of 160–175 °C and a screw speed of 60 rpm to obtain a masterbatch of 6 wt% mono-filler nanocomposites. The bi-filler hybrids with 6 wt% GNP/MWCNT content with varying ratios of fillers (4.5/1.5, 3/3 and 1.5/4.5) were prepared by mixing the two mono-filler composites in appropriate proportions in a second extrusion run. All compounds used in this study were processed in two extrusion runs and pelletized at the end. Table 2 presents the nanocomposites produced for this study.

The CAD modeled test samples of length 20 mm, width 10 mm and thickness 2 mm were 3D printed from pellets using a 3D printer Ender 5 pro (FDM, Creality 3D Technology Co., Ltd., Shenzhen, China) with a pellet extruder print head v4 MAHOR-XYZ with a printing nozzle of diameter *D* = 0.8 mm. The optimal printing parameters were obtained at a print temperature *T*_p_ = 260 °C, printing speed *V*_p_ = 17 mm/s, layer thickness *L* = 0.2 mm and printing density 100%. The magnetic build platform, consisting of a flexible steel plate coated with a PEI (Polyetherimide) surface to enhance 3D print adhesion, was heated to T_bp_ = 100 °C, and a glue stick was applied to further improve adhesion. The samples were printed line by line in a direction parallel to the sample length, with a raster angle of 0°.

Samples for thermal and electrical characterization were prepared with different dimensions (as reported in the respective subsections) to accommodate the specific holders and measurement setups of the respective instruments. As thermal and electrical conductivity are intrinsic material properties, these variations in sample size do not affect the reported results.

### 2.3. Morphological Analysis

Transmission electron microscopy (TEM) analysis was carried out using a high-resolution scanning transmission electron microscope (HR-STEM, JEOL JEM 2100, JEOL Ltd., Tokyo, Japan), operated at an accelerating voltage of 200 kV. Graphene nanoplatelets (GNPs) in powder form were first ultrasonically dispersed in ethanol to achieve a homogeneous suspension. A small aliquot of the dispersion was subsequently deposited onto standard copper TEM grids and allowed to dry prior to observation.

The distribution and morphology of nanofillers embedded in the PVDF-based nanocomposites were also thoroughly analyzed through scanning electron microscopy (SEM) to gain insight into their structural organization and dispersion quality. For this purpose, a Tabletop SEM (Mod. SH-4000MB, Hiirox Europe, Limonest, France) was utilized, equipped with a variable accelerating voltage ranging from 1 kV to 30 kV and capable of reaching a maximum magnification of 60,000×. These features enabled high-resolution imaging of the nanocomposite cross-sections, facilitating the identification of filler agglomerates, interfacial adhesion, and the overall homogeneity of the filler distribution.

### 2.4. Thermal Analysis

Specimens with dimensions of approximately 10 mm × 10 mm and a thickness of 2 mm were characterized at room temperature (~25 °C) using the Laser Flash Technique (LFA 467 HyperFlash, Netzsch, Selb, Germany). Prior to measurement, both surfaces of the samples were coated with graphite in order to improve the accuracy and reliability of the thermal response by ensuring uniform absorption of the laser pulse and consistent emission of the thermal signal. Three consecutive measurements were performed for each material under investigation, and the reported values represent the corresponding averages.

The specific heat capacity was determined by the comparative method described in ASTM E1461-2011 [29], with the LFA system calibrated using a certified Cp reference standard (Pyroceram, 10 mm × 10 mm × 2 mm). Sample density at room temperature was obtained via the Archimedes method based on buoyancy. The Laser Flash Technique is widely recognized for its rapid and non-destructive nature, as well as for its high precision in determining thermal diffusivity, especially in small-scale or heterogeneous samples. Further details on the underlying measurement principles and methodological considerations can be found in our previous works [30,31].

### 2.5. Electrical Characterization

**Figure 1:**
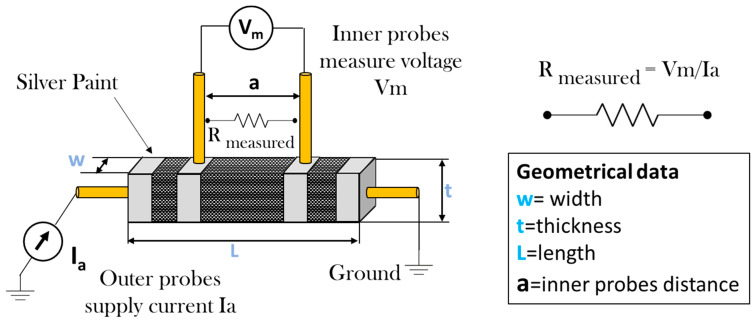
Setup for measuring the DC electrical conductivity with van der Pauw method.

Electrical resistance (*R*) and electrical conductivity (*σ*) at a room temperature of 25 °C were measured according to the van der Pauw method. Figure 1 illustrates the experimental setup employed to measure the electrical conductivity of the samples using the four-probe method, a highly accurate and widely recognized technique for resistance measurement. In this configuration, four electrical probes are positioned along the length of the sample. Two outer probes are used to supply current (Ia) across the sample, while the two inner probes are utilized to measure the voltage drop (Vm) induced by the current flow. This method eliminates the influence of contact resistances at the current injection points, ensuring precise measurement of the sample’s intrinsic resistance (R_measured_). The electrical conductivity (σ) is subsequently calculated using Ohm’s law in its second form, which relates the resistance to the sample’s geometrical dimensions:

(1) $$\sigma =\frac{1}{R_{measured}}\cdot \frac{a}{w\cdot t}$$

where a is the length [m] of the sample between the inner voltage probes, w is the width [m] of the sample, t [m] is the thickness of the sample.

Furthermore, to guarantee ohmic contact and minimize any additional contact resistance at the interfaces between the sample and the measurement electrodes, the electrodes are coated with silver paint with a thickness of about 50 mm and a volume resistivity of 0.00001 Ω·m (LOCTITE^®^ 3863 Circuit+^TM^, Henkel, Düsseldorf, Germany). This ensures a uniform current distribution and accurate resistance measurement.

The dimension of the test section ~20 mm × 10 mm × 2 mm^3^ was measured for each sample by a digital micrometer with an accuracy of 0.001 mm. Three samples were electrically characterized, and the average values were reported. The resistive heating test (Joule heating) was performed by an experimental setup consisting of a power supply providing the voltage and measuring the current (GW Instek, New Taipei City, Taiwan) and a thermocouple type K connected to Keithley 6517B (Keithley Instruments, Solon, OH, USA). To perform the test, electrical contacts of copper wire fixed with silver glue were realized on the short sides of the sample and connected to the power supply. Thus, the tested section of the sample was of length L = 20 mm, width W = 10 mm and thickness t = 2 mm. Applying voltage of 2 V, 3 V and 4 V to the sample, the local temperature evolution over time was measured by a thermocouple positioned at the center of the sample surface and recorded. Data for the applied voltage, temperature, current, and time during the four heating and cooling cycles were collected and recorded by the Lab View software (version 18.0). Before testing the Joule heating, the initial resistance of the sample was measured by a Keithley 6517B.

### 2.6. Design of Experiments

**Figure 2:**
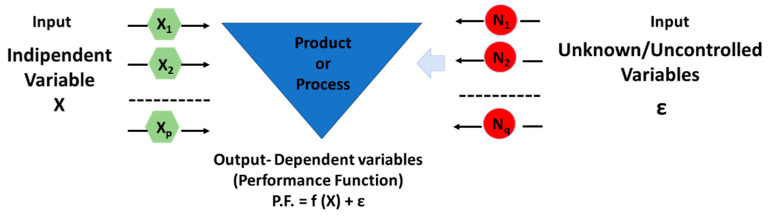
Schematic view of the Experiment Design.

The methodology of experimental design (DoE) conceptualizes a system under study (as illustrated in Figure 2) as a “black box” where specific inputs, represented by independent variables, are manipulated to observe their influence on outputs, defined as dependent variables (YYY). By systematically regulating these inputs and monitoring the corresponding changes in outputs, the technique aims to discern the impact of the controlled variables while mitigating interference from extraneous or unaccounted-for factors [32]. This structured approach facilitates a precise understanding of how variations in the input parameters affect the resulting outcomes, thereby enabling the identification of optimal parameter configurations to enhance the desired performance or characteristics of the system.

This research utilizes the Design of Experiments (DoE) methodology to analyze the influence of two nanofiller concentrations, specifically the weight percentages of Multi-Walled Carbon Nanotubes (MWCNTs) and Graphene Nanoplatelets (GNPs), on the experimentally measured electrical (σ) and thermal conductivities (λ).

To effectively implement DoE, it is crucial to define a suitable number of values for the input variables. In this investigation, the vector of input variables, $\bar{x}$ has been specified as follows:

(2) $$\bar{x}=({wt\%}_{MWCNTs},{wt\%}_{GNs})\epsilon R^{2}$$

within the following values admissible for each carbon-based filler, respectively:

(3) $${{wt\%}_{MWCNTs}}_{1}=0,{{wt\%}_{MWCNTs}}_{2}=1.5,{{wt\%}_{MWCNT}}_{s3}=3,{{wt\%}_{MWCNT}}_{s4}=4.5,{{wt\%}_{MWCNT}}_{s5}=6$$

(4) $${{wt\%}_{GNs}}_{1}=0,{{wt\%}_{GNs}}_{2}=1.5,{{wt\%}_{GN}}_{s3}=3,{{wt\%}_{GN}}_{s4}=4.5,{{wt\%}_{GN}}_{s5}=6$$

Consequently, the variable space, denoted as the compact set D, is mathematically expressed in the following manner:

(5) $$D={wt\%}_{MWCNT}\times {wt\%}_{GN}\subset R^{2}$$

while the dependent variable (i.e., the electrical and thermal conductivity) is assessed for the following ordered pair $\left({wt\%}_{MWCNTs},{wt\%}_{GNs}\right)$ of the input variable vector, i.e.,:

(6) $$\left(0,6\right),\;\left(1.5,4,5\right),\left(3,3\right),\left(4,5,1.5\right),\left(6,0\right)\;\epsilon \;D.$$

For the sake of methodological transparency, it is important to point out that a One Factor At a Time (OAT) approach was used to assess the influence of input parameters on the performance function. Each parameter was varied independently between its minimum and maximum values while the others were held constant. Main effect coefficients and plots summarize the influence of each parameter, allowing identification of the most significant factors. Since each condition was evaluated once and no stochastic noise was included, formal replicates and ANOVA (Analysis Of Variance) were not performed; variability is represented by the range of observed outputs.

### 2.7. Response Surface Method (RSM)

Response Surface Methodology (RSM), first introduced by Box and Wilson in the early 1950s, continues to serve as a widely applied computational framework rooted in the principles of Design of Experiments (DoE) [33,34]. This approach is employed to predict the correlation between multiple input parameters and their associated experimental outcomes. When the exact formulation of the performance function (P.F.) is unknown, RSM endeavors to approximate the response surface (R.S.), with the objective of identifying regions where the output achieves optimal values as the input factors are varied. Typically, the response surface is expressed in the following format:

(7) $$R.S.=f\;\left(X_{1},{\;X}_{2},\;\ldots X_{n}\right)+\varepsilon$$

In this framework, *f* denotes the mathematical representation of the relationship between the response surface (R.S.) and the independent input factors (X_i_), while ε captures the experimental error, assumed to follow a normal distribution with zero mean and constant variance. To model the behavior of the surface, polynomial equations are commonly employed; either first-order (linear) or second-order (quadratic) models are generally adequate for analyzing performance across a variety of scenarios. This method proves particularly effective when the response is influenced by two or three input parameters, as is the case in our study, where the outcome (electrical and thermal conductivities) depends on both weight amounts of one-dimensional filler and two-dimensional filler, specifically MWCNTs wt% and GNPs wt%.

Mathematically, the quadratic polynomial model (n = 2) can be represented by the following equation:

(8) $$R.S.=\beta_{0}+\sum_{i=1}^{n}\beta_{i}x_{i}+\sum_{i=1}^{n}\beta_{ii}x_{i}^{2}+\sum_{i=1}^{n-1}\sum_{j=i+1}^{n}\beta_{ij}x_{i}x_{j}$$

In this equation, x_i_ and x_j_ represent the independent input variables, β_0_ is the intercept coefficient, and β_i_, β_ii_, and β_ij_ correspond to the coefficients for the linear, quadratic, and interaction terms, respectively. These coefficients are calculated using the least squares method.

### 2.8. Theoretical Model for the Electrical Heating

The time-dependent temperature rises in our systems subjected to Joule (ohmic) heating are theoretically evaluated by applying the following equation [35]:

(9) $$C\frac{dT}{dt}=P-Sh\left(T-T_{ext}\right)$$

The left-hand side, CdT/dt, represents the rate of change in the system’s thermal energy, where C is the heat capacity in J/K). On the right-hand side, P denotes the power (in W) generated by electrical heating (Joule effect, P = V^2^/R where is the voltage applied to sample having electrical resistance R) while Sh(T − T_ext_) represents the convective heat losses to the surroundings (S is the exchange area in m^2^) proportional to the difference between the system temperature T (in K) and the external temperature T_ext_ (in K) through the heat transfer coefficient (*h*, measured in W/m^2^K). Thus, the equation captures the balance between heat generation and heat dissipation, allowing prediction of the temperature evolution over time.

The solution of this first-order heat balance differential equation leads to the following relationship used for our theoretical prediction:

(10) $$T\left(t\right)=T_{ext}+\frac{P}{Sh}(1-e^{-\frac{t}{\tau }})$$

where τ is a time constant defined as C/Sh.

The accuracy of these predictions, offering insights into thermal performance in real-world applications, is validated against experimental and simulation data.

The validity of adopting an exponential model to describe the transient thermal behavior under convective boundary conditions is supported by the Biot number (Bi), which provides a dimensionless measure of the ratio between internal thermal resistance within the solid and the external convective resistance at the surface. The Biot number is calculated as [36]:

(11) $$B_{I}=\frac{hL_{c}}{\lambda }$$

where h is assumed 10 W·m^−2^·K^−1^, which is a representative value for convective heat transfer, L_c_ is the characteristic length of the sample (defined as the ratio between its volume and surface area, which for thin geometries is often approximated by half the thickness, i.e., 1 mm in the present study), and λ is the thermal conductivity of the material determined experimentally from our measurements (0.304 W·m^−1^·K^−1^ for the sample 6GNP/PVDF). Substituting these values yields a Biot number of approximately 0.06.

Since this Biot number falls below the critical threshold of 0.1, the internal thermal resistance of the solid is negligible compared to the convective boundary layer.

For the remaining formulations such condition is well satisfied. Only for the composite (namely 6MWCNT/PVDF) with the lowest thermal conductivity does the Biot number approach the upper limit of 0.1, remaining marginally within the acceptable range.

This condition substantiates the validity of assuming a spatially uniform temperature within the solid domain. Consequently, the adoption of a lumped-capacitance model and its characteristic exponential temperature decay is fully justified, as it appropriately captures the transient thermal response under convective heat transfer.

Radiative heat losses were not considered in this study for any of the formulations. This assumption is justified because, even in the worst-case scenario (6 GNP/PVDF at the maximum applied voltage of 4 V), where the sample temperature reaches around 88 °C, the radiative loss estimated using the Stefan–Boltzmann law, is approximately 0.093 W, representing only about 10% of the dissipated electrical power (0.89 W). As reported in Section 3.3 Joule Heating Characteristics for all other formulations and conditions, temperatures remain below 80 °C, making radiation an even smaller contribution. Therefore, radiative heat transfer can be considered negligible compared to conduction, and the theoretical temperature curves closely match the experimental measurements as reported in Section 3.6.1 Model Validation.

### 2.9. Simulation Study on the Electrical Heating and De-Icing Properties

Simulation studies focusing on the Joule heating effect and the de-icing performance of the most thermally efficient formulation, namely 6GNP/PVDF, were carried out using the commercial software COMSOL Multiphysics^®^ (Version 6.1), which is based on the Finite Element Method (FEM).

The primary objective of these simulations was to perform a detailed numerical-to-experimental/theoretical comparison in order to validate the computational model. The validation phase was essential to ensure that the model could accurately reproduce the physical behavior observed experimentally. Once validated, the model was subsequently employed to investigate the de-icing performance of the material under various conditions, aiming to assess its effectiveness and potential for practical applications.

**Figure 3:**
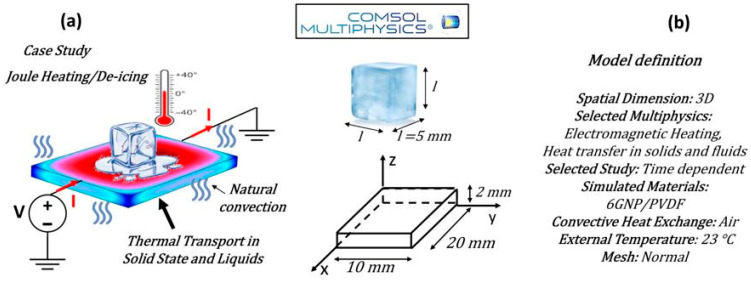
Case study addressed in the present work in (**a**) and key model definition adopted in the simulation in (**b**).

A schematic representation of the simulated system, along with the definition of the case study, is presented in Figure 3a,b, respectively.

The main modeling assumptions, governing equations, boundary conditions, and material properties are described in detail within the study.

Starting from the first law of thermodynamics and assuming constant pressure conditions, the thermal energy conservation equation governing heat conduction in a solid can be derived. In Cartesian coordinates, and considering an infinitesimal control volume ∆_x_∆_y_∆_z_, the equation takes the following form [35]:

(12) $$\frac{\partial }{\partial x}\left(\lambda \frac{\partial T}{\partial x}\right)+\frac{\partial }{\partial y}\left(\lambda \frac{\partial T}{\partial y}\right)+\frac{\partial }{\partial z}\left(\lambda \frac{\partial T}{\partial z}\right)+{\left.Q\right|}_{Joule\;heating}=\rho c_{p}\frac{\partial T}{\partial t}$$

In the above equation, ρ denotes the density of the material [kg·m^−3^], c_p_ represents the specific heat capacity [J·kg^−1^·K^−1^], and λ corresponds to the intrinsic thermal conductivity [W·m^−1^·K^−1^]. The internal heat generation term, associated with Joule heating, is defined as Q = J·E, where J indicates the current density [A·m^−2^] and E is the electric field strength [W·A^−1^·m^−1^], arising from the application of different voltage levels (2 V, 3 V, and 4 V). The initial and boundary conditions required for the resolution of the thermal energy balance (Equation (1)) are systematically presented in Table 3.

The other physical parameters were selected based on the experimental findings presented in this study. Specifically, the electrical and thermal conductivity values are 55.80 Sm^−1^ and 0.304 Wm^−1^·K^−1^, respectively, the specific heat capacity is 1220 J·kg^−1^·K^−1^, and the density is assumed to be 1757 kg·m^−3^. Finally, Table 4 provides a summary of the thermal properties of water in its solid and liquid phases, reported at representative temperatures of −8 °C and 27 °C, respectively. The latent heat of diffusion is 333.5 kJ/kg and the ice cube is initially at −20 °C.

## 3. Results and Discussions

This section reports on a thorough morphological analysis conducted to examine in detail the intrinsic characteristics of the fillers employed and their subsequent dispersion within the polymer matrix. Building on these observations, the experimental investigation explores the electrical and thermal conductivities of the composites across various filler loadings. A Design of Experiments (DoE) approach combined with Response Surface Methodology (RSM) was further applied to statistically evaluate the influence of filler content on the electrical and thermal performance of the composites. Additionally, a multiphysics simulation is employed to complement the experimental data. Once validated against both experimental results and theoretical expectations, the simulation offers valuable insight into the thermal behavior of the composites, highlighting their potential for real-world applications such as de-icing systems.

### 3.1. Morphological Investigation

**Figure 4:**
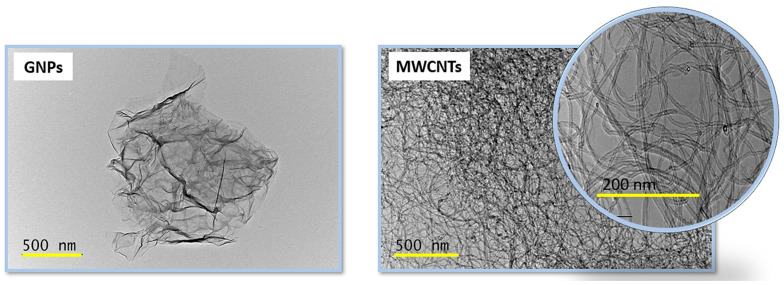
TEM images of the pristine nanofillers: (**left**) graphene nanoplatelets (GNPs) displaying layered, sheet-like structures with extended lateral dimensions and (**right**) multi-walled carbon nanotubes (MWCNTs) exhibiting a high aspect ratio and entangled morphology.

The transmission electron microscopy (TEM) images of Figure 4 provide valuable insight into the morphology of the carbon-based fillers employed in the fabrication of the nanocomposites. The graphene nanoplatelets, shown in the left micrograph, exhibit the typical two-dimensional, sheet-like architecture with lateral dimensions of several hundred nanometers, as indicated by the 500 nm scale bar. Their wrinkled and crumpled appearance highlights the intrinsic flexibility of graphene layers and the abundance of edge sites, features that play a critical role in enhancing the interfacial contact with the polymeric matrix and in promoting the formation of efficient thermal and electrical percolation networks. In contrast, the micrograph on the right reveals the characteristic entangled network of carbon nanotubes, with diameters in the nanometer range and lengths extending to several hundreds of nanometers, as evidenced by the 500 nm scale bar and by the 200 nm magnification. The pronounced one-dimensional tubular structure and high aspect ratio of CNTs, coupled with their entangled morphology, allow them to function as efficient load-bearing elements while promoting the formation of continuous interconnected networks that significantly enhance conductivity and crack-bridging performance in composite systems.

**Figure 5:**
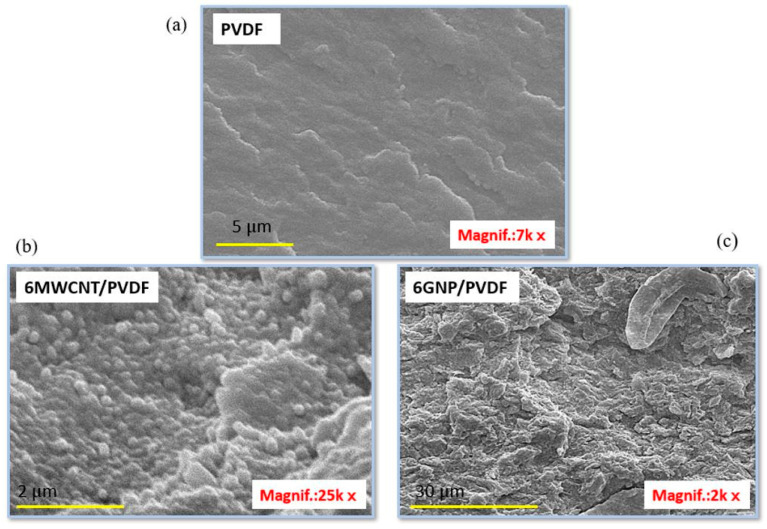
SEM images of the neat PVDF (**a**) and the PVDF-based nanocomposites: (**b**) 6MWCNT/PVDF, showing an encapsulated nanotube network, and (**c**) 6GNP/PVDF characterized by a uniform and stratified presence of graphene nanoplatelets embedded within the PVDF matrix.

The SEM micrographs in Figure 5 illustrate the surface morphology of neat PVDF and PVDF-based nanocomposites incorporating different carbon nanofillers. The neat PVDF in Figure 5a shows a typical plastic morphology characterized by a smooth surface without significant structural details. While in Figure 5b, the SEM micrograph of the 6MWCNT/PVDF system, at the high magnification (25k×), shows a large number of carbon nanotubes encapsulated by the PVDF matrix and protruding from the polymer surface. In contrast, the panel (c) displays the SEM image of the 6GNP/PVDF nanocomposite (2000×), which reveals a rough, stratified morphology with graphene nanoplatelets uniformly distributed within the polymer matrix. This interconnected arrangement suggests strong filler–matrix interactions, providing both mechanical reinforcement and continuous pathways favorable for electrical and thermal transport.

**Figure 6:**
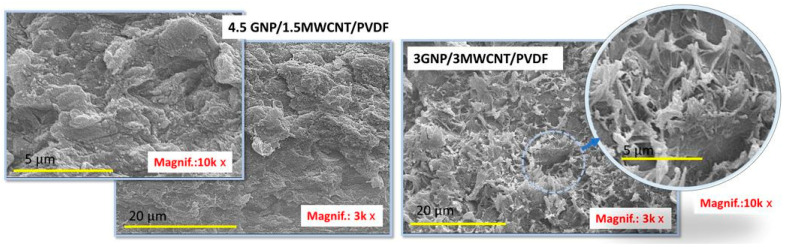
SEM images of the hybrid systems based on: (**left panel**) 4.5GNP/1.5/MWCNT/PVDF, highlighting the formation of a dense structure, and (**right panel**) 3GNP/3MWCNT/PVDF with a more open hybrid architecture.

Finally, Figure 6 presents SEM micrographs of the hybrid systems: (left panel) the 4.5GNP/1.5MWCNT/PVDF and (right panel) 3GNP/3MWCNT/PVDF, acquired at lower magnification (3000×) and higher magnification (10,000×) to provide both an overview of the morphology and finer structural details.

Here, the morphology is significantly more open and porous, with visible plate-like GNP structures intercalated within the nanotube network. The synergistic integration of one-dimensional MWCNTs and two-dimensional GNPs usually creates a more interconnected and tortuous conductive network, likely facilitating enhanced electron transport pathways while also increasing the specific surface area available for polymer–filler interactions. The observed heterogeneity in the filler distribution reflects the balance between nanotube entanglement and graphene sheet stacking, which is critical for balancing the multifunctional performance of hybrid nanocomposites.

### 3.2. Experimental Electrical and Thermal Conductivity Evaluation

**Figure 7:**
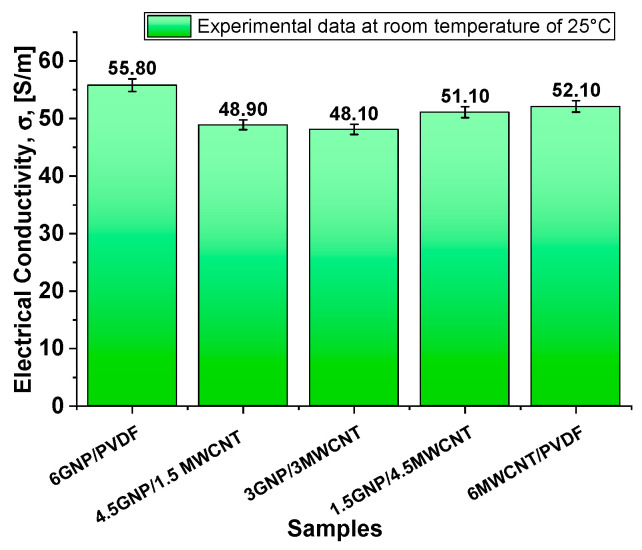
Electrical conductivity of the 3D printed PVDF-composites incorporating GNPs, MWCNTs, and their combinations.

The bar chart of Figure 7 illustrates the electrical conductivity (σ measured in S/m) of PVDF-based composites containing varying formulations of graphene nanoplatelets (GNPs) and multi-walled carbon nanotubes (MWCNTs). The data reveals that composites with higher concentrations of GNPs exhibit significantly better conductivity compared to those containing MWCNTs. Specifically, the composite with 6 wt% GNPs achieves the highest conductivity value, approximately 55.80 S/m, whereas the composite with 6 wt% MWCNTs exhibits a lower conductivity, around 52.10 S/m. Hybrid systems, such as 4.5 wt% GNPs/1.5 wt% MWCNTs and 3 wt% GNPs/3 wt% MWCNTs, show intermediate conductivity values in the range of 48–51 S/m. In our previous study [37], the percolation threshold of MWCNT/PVDF nanocomposites was determined to be between 2 and 3 wt% MWCNTs, while that of GNP/PVDF was slightly below 2 wt% GNPs.

To explain these trends, it is essential to relate the microstructure density and the interactions between the fillers and the PVDF matrix observed on SEM images in Figure 5 and Figure 6 with the mechanisms driving conductivity in such systems.

PVDF is a semicrystalline polymer with strong polarity due to fluorine-induced dipoles, which critically influence filler dispersion. Graphene nanoplatelets (GNPs), with their extended 2D structure, interact effectively with these dipoles, promoting partial polarization, improved dispersion, and reduced aggregation. Their nanoscale thickness ensures deep embedding and strong interfacial contact, minimizing resistance and enabling efficient electron transport—resulting in superior conductivity in GNP-rich systems.

Conversely, MWCNTs exhibit weaker interfacial interactions with PVDF due to their inert 1D structure, leading to aggregation and poor network formation. Their lower contact area and reliance on tunneling conduction are further hindered by electrostatic repulsion in the polar matrix, which increases tunneling distances and limits conductivity.

Hybrid systems such as 4.5GNP/1.5MWCNT, 3GNP/3MWCNT, and 1.5GNP/4.5MWCNT display intermediate conductivities (48.9, 48.1, and 51.1 S/m, respectively), suggesting a non-linear relationship between filler ratio and electrical performance.

In particular, in the 1.5GNP/4.5MWCNT hybrid system, a favorable percolation architecture emerges: the dominant CNT fraction forms a continuous conductive backbone, while the small amount of GNPs acts as interconnecting bridges that reduce inter-tube resistance and facilitate electron tunneling between CNT clusters. This optimized synergistic configuration enhances network connectivity and charge-transfer efficiency, explaining the slightly higher conductivity (≈51 S m^−1^) observed for this hybrid compared to other mixed ratios.

These values indicate partial synergy, where the combination of 2D GNPs and 1D MWCNTs enhances network connectivity, but also highlights the challenge of optimizing dispersion and percolation simultaneously. The slightly lower conductivity of 3GNP/3MWCNT and 4.5GNP/1.5MWCNT, compared to the single-filler systems, suggests that mixed fillers may experience reduced individual percolation efficiency or increased interfacial resistance due to differing morphologies and surface chemistries. Overall, while hybrid fillers can bridge the advantages of GNPs and CNTs, the observed conductivities emphasize the need for precise tuning of filler ratios and interfacial engineering to fully exploit synergistic effects.

**Figure 8:**
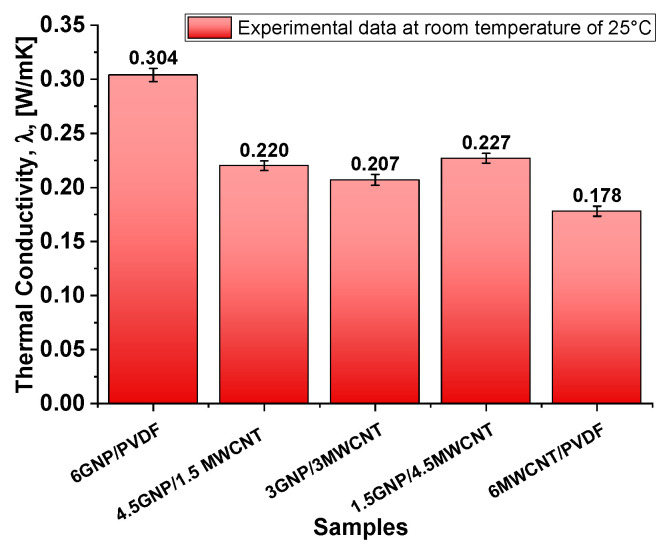
Thermal conductivity of the 3D printed PVDF-composites incorporating GNPs, MWCNTs, and their combinations.

The results presented in Figure 8 highlight the variation in thermal conductivity (λ) among the same nanocomposite samples at room temperature (25 °C). The sample containing only graphene nanoplatelets (6GNP/PVDF) exhibits the highest thermal conductivity at 0.30 W/m·K, indicating the superior heat conduction ability of GNPs within the polymer matrix. In fact, GNPs possess a two-dimensional planar structure with an extremely high intrinsic in-plane thermal conductivity (up to ~5000 W/m·K for pristine graphene), which facilitates efficient phonon transport across the filler network. Moreover, the larger surface area and aspect ratio of GNPs promote better interfacial contact with the polymer matrix, improving thermal percolation pathways. In contrast, the sample comprising only multi-walled carbon nanotubes (6MWCNT/PVDF) shows the lowest thermal conductivity (0.178 W/m·K), suggesting that, under the tested conditions, MWCNTs are less effective in enhancing thermal transport. Most likely, this is because their one-dimensional tubular structure often results in higher interfacial thermal resistance due to limited contact area and greater phonon scattering at the interface.

Once again, the observed behavior of the 1.5GNP/4.5MWCNT hybrid system, which exhibits a slightly higher thermal conductivity (0.23 W·m^−1^·K^−1^), can be attributed to a favorable percolation architecture within the PVDF matrix that facilitates both electron transport and phonon coupling across CNT clusters. This synergistic configuration enhances thermal transport efficiency without excessively disrupting the CNT network continuity, as observed at higher GNP loadings (e.g., 4.5GNP/1.5MWCNT and 3GNP/3MWCNT, with thermal conductivities of 0.22 W·m^−1^·K^−1^ and 0.21 W·m^−1^·K^−1^, respectively), where a more open and porous morphology is evident (see Figure 6, right panel). Overall, this behavior reveals a non-linear dependence on the GNP-to-MWCNT ratio, primarily governed by the resulting microstructural morphology.

Overall, these findings underscore the critical role of both nanofiller type and ratio in determining thermal conductivity. Achieving optimal performance requires careful balancing, as an improper combination of fillers may hinder—rather than enhance—the formation of an effective thermal network.

Moreover, the crystallinity of the GNP and MWCNT fillers, as well as that of the PVDF-based nanocomposites, is a factor influencing the electrical and thermal conductivities of the composites. In our previous study [37], the crystalline structure of these materials was investigated by XRD analysis. A narrower peak was observed for MWCNTs, suggesting a more crystalline structure compared to GNPs. In the nanocomposites containing 6 wt% filler, the PVDF crystallinity decreased from 65% in the neat polymer to 55% with the addition of GNPs and to 47% with the addition of MWCNTs. This decrease reflects strong polymer–filler interactions, which suppress polymer crystallization at the interfaces. The combination of strong interfacial interactions and the higher specific surface area of GNPs compared to MWCNTs (see Table 1) resulted in the formation of a more effective network for electrical and thermal transport in the GNP/PVDF composites than in the MWCNT/PVDF systems.

In conclusion, the correlation between morphology and transport behavior can also be interpreted in terms of the effective inter-filler contact density observed in previous TEM/SEM analyses. The extended overlap of GNP sheets and their higher specific surface area (see the value of SSA in Table 1 and the morphology in Figure 5) promotes dense junction networks and short tunneling paths, resulting in superior electrical and thermal conductivities, equal to 55.8 S m^−1^ and 0.304 W m^−1^ K^−1^, respectively. CNT-based composites, although characterized by fewer contact points, still ensure efficient charge transport but exhibit lower thermal conductivity due to higher interfacial resistance. Hybrid systems (Figure 6) display intermediate compactness, where partial synergy between GNP bridges and CNT backbones enhances overall connectivity while remaining limited by increased interparticle spacing.

### 3.3. Joule Heating Characteristics

**Figure 9:**
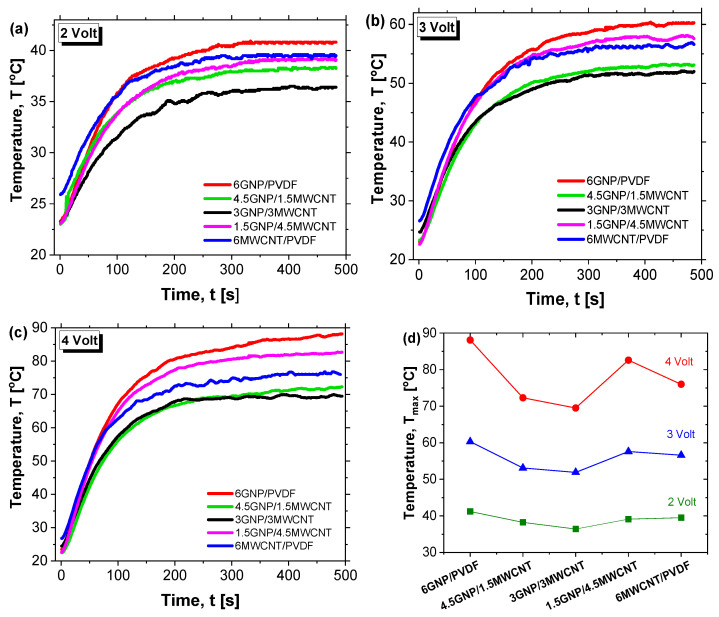
Joule heating temperature vs. time with varying applied voltage: (**a**–**c**) 2 V, 3 V and 4 V; (**d**) Maximal temperature of heating for different compositions with varying the applied voltage 2 V, 3 V, and 4 V.

Figure 9 presents the Joule heating behavior of various nanocomposite formulations under applied voltages of 2 V, 3 V, and 4 V in (a–c), respectively. Among all systems, the composite containing 6 wt% graphene nanoplatelets (6GNP/PVDF) clearly exhibits the best electrothermal performance, reaching the highest temperatures and fastest heating rates at each voltage level. At 4 V, this formulation approaches 90 °C, significantly outperforming all other samples. This superior behavior is attributed to the high electrical and thermal conductivity of graphene nanoplatelets and their ability to form efficient percolating networks within the polymer matrix. The hybrid composites—such as 4.5GNP/1.5CNT and 3GNP/3CNT—show intermediate heating performance. While these systems benefit from the combined conductive properties of both fillers, they fall short of the pure 6% GNP formulation, likely due to suboptimal network formation or filler dispersion that limits thermal transport. These hybrids still outperform the 6% CNT composite, suggesting a synergistic but non-additive effect of the mixed fillers. Notably, the composite containing only carbon nanotubes (6MWCNT/PVDF), while possessing relatively high electrical conductivity—second only to the 6GNP/PVDF system—exhibits the lowest heating efficiency overall. This is explained by its markedly lower thermal conductivity, as previously demonstrated in Figure 8. Despite being electrically conductive, its limited ability to dissipate heat reduces the overall Joule heating performance, highlighting the importance of not just electrical but also thermal conductivity in optimizing electrothermal response. Overall, these results underscore the critical role of filler type and morphology. In other words, although the hybrid composites exhibit an open and interconnected morphology (Figure 6), this structural openness is associated with increased interparticle spacing and partial discontinuities in the conductive paths. These gaps hinder continuous electron and phonon transport, reducing both electrical and thermal conductivities compared to the dense, stacked and planar GNP network in the 6GNP/PVDF sample (Figure 5). Furthermore, in some hybrid formulations, the inhomogeneous dispersion of CNTs and GNPs can limit network efficiency. Similar trends have been reported in our previous study on hybrid CNT/GNP systems, where excessive porosity or incomplete filler contact decreases the effectiveness of thermal percolation despite adequate electrical conductivity [37]. Consequently, the superior performance of the single-filler GNP composite arises from its continuous and compact architecture that minimizes interfacial resistance and enhances both charge and heat transfer.

The 6GNP/PVDF composite stands out as the most effective formulation for applications requiring efficient and rapid electrical heating, while the limited performance of the 6MWCNT/PVDF system reinforces the necessity of a balanced conductive network that supports both charge transport and heat diffusion. Therefore, this most thermoelectrically efficient configuration (6GNP/PVDF) will be further investigated through dedicated theoretical and numerical studies. Moreover, the lack of linearity observed in the behavior of hybrid configurations suggests that a statistical approach using Design of Experiments (DoE) and Response Surface Methodology (RSM) is recommended to gain deeper insights into the complex interactions and optimize the formulation strategy.

The apparent differences in the initial temperature at t = 0 in Figure 9a–c arise from minor local temperature fluctuations or sensor response times.

These slight variations do not affect the reported Tmax, which is primarily governed by the second term in the thermal response model of the previous Equation (10). In this expression, the term (P/Sh)∙(1 – e − t/τ) dominates the temperature evolution, as it reflects the balance between power input, heat dissipation, and the system’s thermal time constant, and, thus, controls the maximum temperature reached. Consequently, T_max_ is strongly influenced by sample composition, thermal conductivity, and heat capacity, rather than minor variations in the initial temperature.

The remaining Figure 9d shows the maximum equilibrium temperature (T_max_) of PVDF-based nanocomposites under applied voltages of 2, 3, and 4 V. Regardless of the applied voltage, the 6GNP/PVDF formulation consistently reaches the highest T_max_, confirming its superior thermal conductivity compared to both hybrid and CNT-based systems.

Table 5 summarizes the values of the Joule heating characteristics on the equilibrium plateau at applied voltages of 2 V, 3 V and 4 V, comparing the effect of compositions.

The generated heat *H* and the electric power *P* at the time (t = 500 s) at which the current is measured were calculated by Joule’s law by Equations (13) and (14), respectively:

(13) $$H=\;I^{2}\cdot R\cdot t\;=\;V\cdot I\;\cdot t$$

(14) $$P=I\cdot V=V^{2}/R$$

where *V* is the applied voltage, *I* is the measured current, *R* = *V*/*I* is the resistance, and *t* is the time at which the current is measured. The values of maximum equilibrium temperature (*T_max_)*, the heating rate (*H_r_ = dT*/*dt*) determined from the slope of the initial linear region within the first 10 s, the heat efficiency, *H_eff_* = (1 − (*T_o_*/*T_max_*)) × 100, in %, where T_o_ = 25 °C (as the initial temperature) are also reported for all tested samples.

### 3.4. Design of Experiment: Dex Scatter Plot and Main Factor Plot

**Figure 10:**
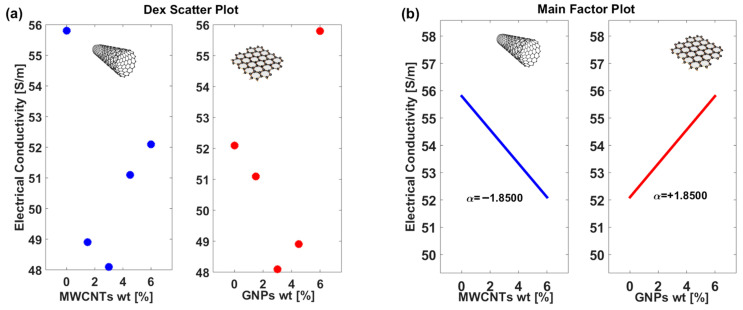
Dex Scatter Plot (DSP) and Main Factor Plot (MFP) for the experimental data of the electrical conductivity [S/m] in panels (**a**) and (**b**), respectively.

The provided Figure 10 illustrates the influence of two different additives, Multi-Walled Carbon Nanotubes (MWCNTs) and Graphene Nanoplatelets (GNPs), on the electrical conductivity of a composite material. Although factors such as dispersion quality and interfacial interactions may influence the overall conductivity, they are not directly quantifiable within the adopted experimental framework; therefore, the DoE model was restricted to compositional variables, which represent the only systematically controllable parameters. The data is presented in two forms: scatter plots to visualize the raw trends (panel (a)) and main factor plots to quantify the primary effects (panel (b)).

In Figure 10a, the scatter plots reveal contrasting behaviors of the two additives. For MWCNTs, the electrical conductivity exhibits a clear downward trend as their weight percentage increases, suggesting a negative correlation. Conversely, for GNPs, the electrical conductivity increases with their weight percentage, indicating a positive correlation. These trends highlight the fundamentally different roles of MWCNTs and GNPs in modifying the electrical properties of the composite.

Figure 10b provides a more quantitative perspective through main factor plots, where linear trends summarize the primary effects of the two factors on electrical conductivity. Specifically, the α values—derived from the slopes of the trend lines—serve as quantitative indicators of how sensitively the composite’s electrical conductivity responds to changes in filler loading. For MWCNTs, the slope of α = −1.8500 confirms the strong negative impact observed in the scatter plot, suggesting that increasing the MWCNT content reduces the conductivity significantly. On the other hand, the slope for GNPs, α = +1.8500, indicates an equally strong but opposite effect, where increasing the GNPs content enhances conductivity. The slopes’ equal magnitudes suggest that the two factors exert comparable influences on conductivity, albeit in opposing directions.

**Figure 11:**
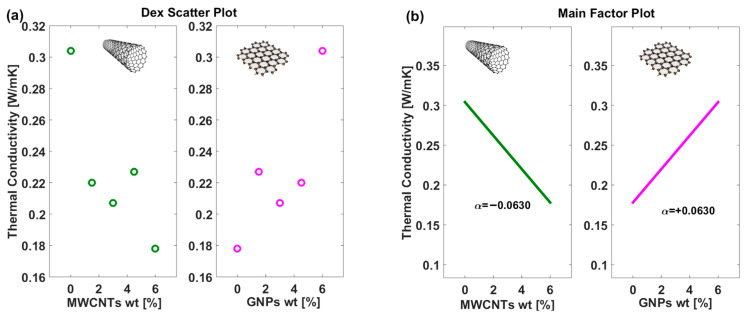
Dex Scatter Plot (DSP) and Main Factor Plot (MFP) for the experimental data of the thermal conductivity [W/mK] in panels (**a**) and (**b**), respectively.

It is important to point out that, as described in Section 2.6 (Design of Experiments), a One Factor At a Time (OAT) approach was employed to isolate the effect of each filler. While the Main Factor Plots effectively capture these single-factor effects, providing a systematic understanding of each filler’s influence, hybrid samples can deviate from these trends due to synergistic interactions between MWCNTs and GNPs. For instance, in the 1.5GNP/4.5MWCNT formulation (see previous Figure 7), increasing MWCNT content experimentally leads to higher electrical conductivity, reflecting the formation of a more efficient interconnected network beyond the predictions of single-factor analysis. Nonetheless, the DoE framework remains a powerful tool, identifying key factors and their relative contributions, and providing a robust foundation for interpreting complex hybrid behaviors. Similarly, Figure 11a,b presents the Dex Scatter Plot (DSP) and the Main Factor Plot (MFP), respectively, for the experimental thermal conductivity data [W/mK] obtained by varying the weight percentage of multi-walled carbon nanotubes (MWCNTs) and graphene nanoplatelets (GNPs).

Once again, the distinct clusters in the scatter plot highlight the different thermal transport behaviors associated with each filler type, reflecting their morphological and interfacial properties within the composite matrix. In particular, in Figure 11b, the main factor plot not only illustrates the overall trend of thermal conductivity with varying filler content but also quantifies the effect of each filler type through the reported α values. Notably, the slopes of the trend lines are reversed between the two filler types, which emphasizes the distinct thermal behaviors of the composites. For example, the GNP-based composites exhibit a positive slope—reflected in a higher α value (+0.0630)—indicating that thermal conductivity increases markedly with an increase in filler loading. In contrast, the MWCNT-based composites show a reversed slope, suggesting a relatively diminished enhancement in thermal conductivity per unit increase in filler content. This reversal of slopes not only underscores the fundamentally different heat transfer mechanisms associated with each filler type—likely due to variations in morphology, interfacial thermal resistance, and network connectivity—but also highlights the importance of selecting the appropriate filler to optimize composite performance. The numerical α values further reinforce these differences, providing a robust, quantitative basis for tailoring composite formulations, highlighting that graphene nanoplatelets are far more effective in enhancing the heat conduction compared to multi-walled carbon nanotubes. In summary, these results demonstrate that the incorporation of GNPs is beneficial for enhancing both electrical and thermal conductivity, whereas MWCNTs appear to suppress it under the conditions studied. The insights provided by this analysis can inform the optimization of composite formulations, where a balance between MWCNTs and GNPs may be necessary to achieve desired electrical properties. Further investigation into the interaction effects between the two factors or other contributing variables could provide additional guidance for tailoring the material’s performance.

### 3.5. Response Surface Method

The statistical methodology known as Response Surface Methodology (RSM) is utilized in our investigation to develop an analytical expression (Equation (8), as previously presented) that accurately reflects the relationship between the key physical properties—such as electrical and thermal conductivity—obtained through experimental tests. These properties are examined with respect to the changing weight concentrations of multi-walled carbon nanotubes (wt% MWCNTs) and graphene nanoplatelets (wt% GNPs). This technique offers significant insights into optimizing the composite’s structural configuration for improved performance.

In the current investigation, the functions of interest for Response Surface Methodology (RSM) are defined by the physical properties experimentally measured: electrical conductivity (σ) and thermal conductivity (λ). To generalize, we introduce the term “physical property” (PP), which applies to both properties. The independent variables are represented by the weight percentage amounts of multi-walled carbon nanotubes (${wt\%}_{MWCNTs}$) and graphene nanoplatelets (${wt\%}_{GNPs}$). Consequently, the objective of RSM is to establish a robust analytical relationship between the specified physical properties (PP) and the defined independent variables: PP = f (${wt\%}_{MWCNTs}$,$\;{wt\%}_{GNPs}$) = f (x_1_,x_2_) for a more compact and appropriate mathematical formulation. Based on Equation (7), the quadratic polynomial that approximates the physical property function *PP* is expressed as:

(15) $$\begin{array}{c} PP=f\left({wt\%}_{MWCNTs},\;{wt\%}_{GNPs}\right)=\\ =f\left(x_{1},x_{2,}\right)=\beta_{0}+\beta_{1}x_{1}+\beta_{2}x_{2}+\beta_{12}x_{1}x_{2}+\beta_{11}x_{1}^{2}+\beta_{22}x_{2}^{2} \end{array}$$

**Figure 12:**
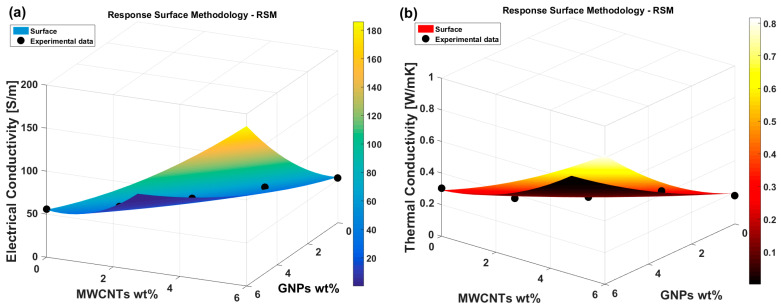
3D-response surface plots for the electrical conductivity (**a**) and thermal conductivity (**b**) as function of the two-weight amount of carbon-based filler, i.e., MWCNts and GNPs.

Figure 12 presents a three-dimensional plot based on the Response Surface Methodology (RSM) that illustrates, respectively, in panel (a) and panel (b), the relationship between the electrical and thermal conductivity and the two independent variables: the weight percentages of multi-walled carbon nanotubes (MWCNTs) and graphene nanoplatelets (GNPs). The surfaces are shown in a gradient color map, with the electrical conductivity (measured in S/m) or the thermal conductivity (in W/mK) represented on the vertical axis, while the horizontal axes correspond to the weight percentages of MWCNTs and GNPs. The color gradient bar on the right indicates the range of electrical and thermal conductivity values.

The plots include experimental data points marked as black dots, allowing for a visual comparison of the model’s predictions and the actual experimental values. These points are scattered across the surfaces, demonstrating how well the fitted surfaces represent the observed data. The surfaces themselves suggest a non-linear relationship between the two independent variables and electrical or thermal conductivity, with the highest conductivity values occurring at certain combinations of MWCNTs and GNPs concentrations. All the coefficients of the two polynomials for the response surfaces related to electrical and thermal conductivity are explicitly listed in Table 6.

These graphical representations help to visually assess the accuracy of the RSM model in predicting electrical and thermal conductivity across different combinations of the two nanofillers. The surfaces provide insights into how the concentration of MWCNTs and GNPs impacts the electrical or thermal conductivity, which is crucial for optimizing the composite materials for enhanced electrical performance.

### 3.6. Simulation Results

This section presents multiphysics simulation results examining the influence of the number and orientation of graphene platelets within the matrix on the thermal properties of the resulting materials. The analysis focuses on heat flux, thermal conductivity, and the spatial-temporal temperature distribution, offering insights into the heat transfer mechanisms governing these composites.

#### 3.6.1. Model Validation

**Figure 13:**
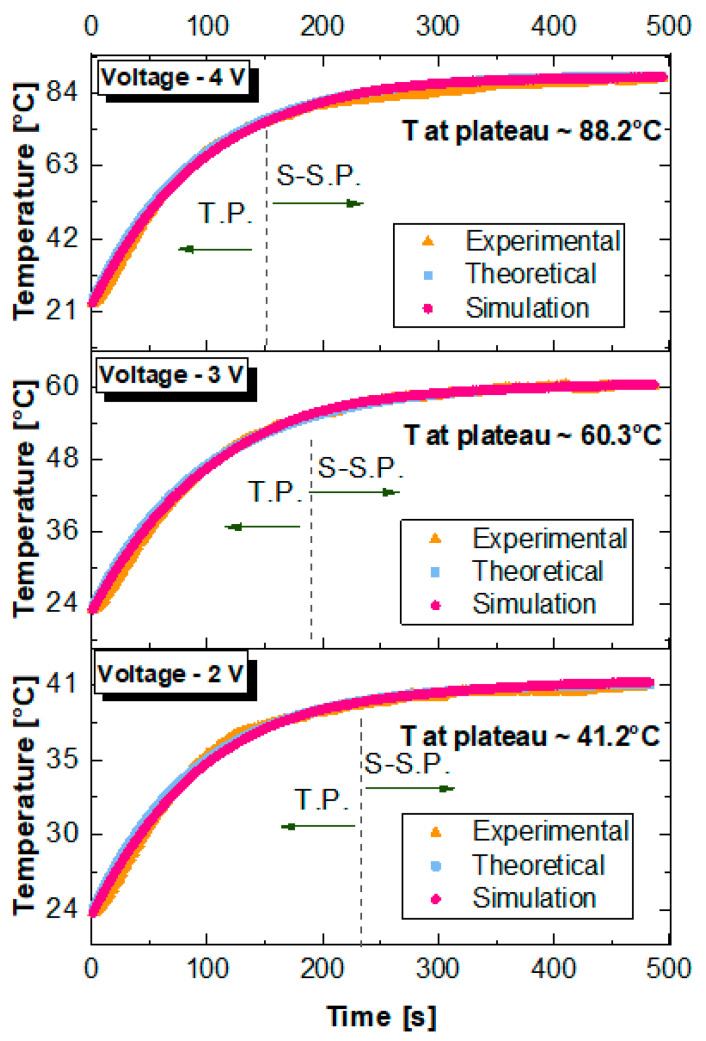
Comparison between experimental data, theoretical model predictions, and numerical simulation results for temperature evolution over time, under three different applied voltages: 2 V (**bottom**), 3 V (**middle**), and 4 V (**top**). Each curve shows the temperature rise, highlighting the transient phase (T.P.) and the transition toward the steady-state plateau (S.-S.P.).

Figure 13 compares the temporal evolution of the surface temperature of the composite samples subjected to Joule heating at applied voltages of 2 V, 3 V and 4 V. Experimental data are benchmarked against predictions from a thermal circuit model and finite element simulations performed with COMSOL Multiphysics. Across all cases, the heating curves exhibit two characteristic phases: an initial transient phase (T.P.), where the temperature rises sharply, followed by a steady-state plateau (S.-S.P.). At 2 V, the system reaches a steady temperature of approximately 41.2 °C, increasing to 60.3 °C at 3 V, and further to around 90 °C at 4 V. This trend aligns with the expected quadratic dependence of Joule heating on the applied voltage. A progressive reduction in the duration of the transient phase with increasing voltage is also observed, indicating that higher applied voltages accelerate the achievement of thermal equilibrium. This behavior is consistently captured by both the theoretical and simulation models. Theoretical and simulation results closely reproduce the measured temperature profile throughout the entire duration of the test, thus confirming the validity of the modeling assumptions and the robustness of the numerical approach. Minor deviations between experimental and predicted curves, primarily in the early transient, can be attributed to experimental uncertainties and to idealized assumptions made in the models, such as homogeneous heat generation and perfect thermal contact conditions. Table 7 presents a comparison between the simulated and experimental plateau temperatures (T_p_) obtained at different applied voltages. The numerical predictions are in excellent agreement with the experimental measurements, showing only minimal percentage deviations, with a maximum difference of 0.898% in the least favorable case. This close correspondence between theoretical predictions and numerical simulations confirms the reliability of the proposed 3D models and further supports the interpretation of the experimental results.

In summary, the excellent fit between experimental, theoretical, and simulation results strongly supports the reliability of the proposed methodology for analyzing and predicting the thermal behavior of polymer composites under electrical stimulation and highlights its potential for guiding the design and optimization of polymer-based thermal management systems. In particular, the combined use of a thermal circuit model and finite element analysis proves to be a powerful strategy: while the thermal circuit model provides rapid, physically intuitive insights into anisotropic heat transfer phenomena, the more comprehensive finite element simulations offer the capture of the detailed three-dimensional spatial distribution of thermal phenomena within the material as depicted in the next subsection.

#### 3.6.2. Temperature and Heat Flux Profiles Within the Sample

**Figure 14:**
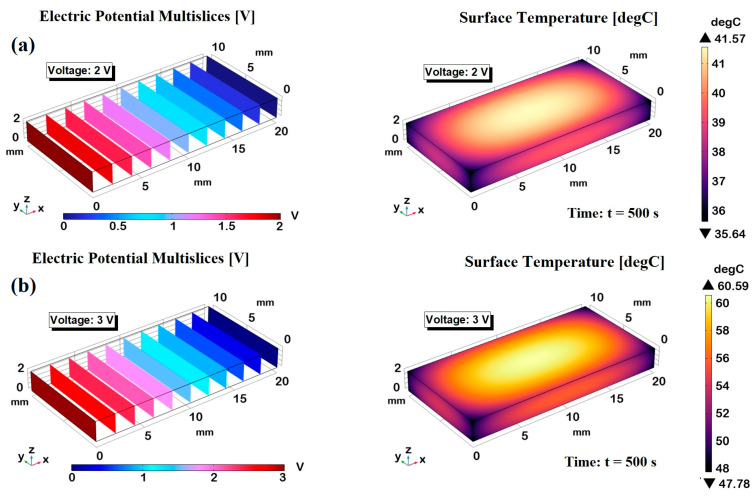

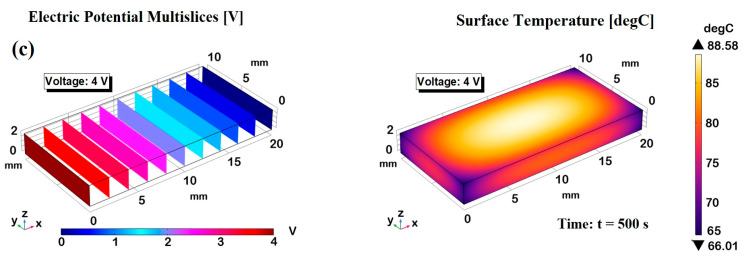
Electric potential profiles (**left panels**) along the direction of applied voltages—2 V, 3 V, and 4 V in (**a**), (**b**), and (**c**), respectively. The corresponding **right panels** present three-dimensional surface temperature distribution induced by Joule heating at t = 500 s.

Figure 14 illustrates the electro-thermal response of graphene-based nanocomposites (6 wt%) subjected to applied voltages of 2 V in panel (a), 3 V in panel (b), and 4 V in panel (c), as modeled via finite element simulations. The left-hand panels show the electric potential distributions, while the right-hand panels display the corresponding three-dimensional steady-state temperature fields, supporting and extending the experimental findings with high predictive accuracy.

As expected for an ohmic medium with homogeneous electrical properties, the electric potential increases linearly along the x-axis, with minimum and maximum values localized at 0 mm and 20 mm, respectively. The uniformity of the potential across transversal cross-sections further confirms the isotropy of the electrical behavior within the plane orthogonal to the voltage direction. Crucially, the simulations predict surface temperature plateaus of approximately 41.2 °C, 61.3 °C, and 90 °C for applied voltages of 2 V, 3 V, and 4 V, respectively, aligning closely with the experimental measurements. This high level of agreement validates the robustness of the numerical model and its underlying assumptions. Furthermore, the 3D thermal maps reveal a clear spatial temperature gradient: the highest temperatures are concentrated at the center of the sample, while peripheral areas—particularly the edges and corners—remain comparatively cooler due to enhanced thermal dissipation mechanisms and boundary effects. This highlights the importance of three-dimensional modeling for accurately capturing the non-uniform thermal fields in electrically activated nanostructured composites. Overall, the multiphysics simulation offers critical insight into the coupled electrical and thermal phenomena governing the system’s behavior.

**Figure 15:**
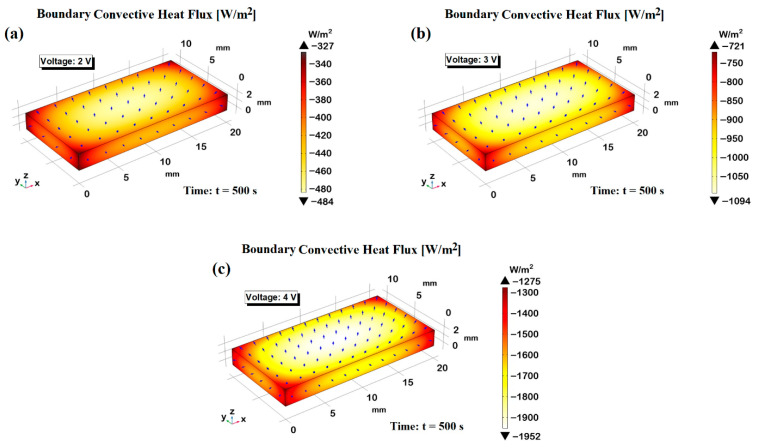
Comparison of convective heat flux distributions in still air under applied voltages of 2 V (**a**), 3 V (**b**), and 4 V (**c**). The arrows represent the direction and relative intensity of the convective flow.

Figure 15 illustrates the simulated convective heat flux distributions in still air for three applied voltage levels: 2 V, 3 V, and 4 V in panels (a–c), respectively. Each subplot displays a top-down view of the heated surface, with a color gradient representing the magnitude of the convective heat flux (in W/m^2^), and superimposed arrows indicating the direction and relative intensity of the air flow resulting from the induced thermal gradients. As the applied voltage increases from 2 V to 4 V, a marked enhancement in convective heat flux is observed, both in magnitude and spatial distribution. At 2 V, the convective flow remains relatively weak and localized, with peak heat flux values around −484 W/m^2^. the air flow arrows remain short and sparsely distributed, reflecting limited thermal-driven motion in the surrounding air. Increasing the voltage to 3 V results in a significantly stronger and more uniform flow, reaching a maximum heat flux of approximately −1094 W/m^2^. The flow vectors become more elongated and denser, indicating enhanced air movement and improved heat removal from the surface. At 4 V, the convective activity intensifies further, with peak values nearing −1962 W/m^2^ and a visibly denser vector field, indicating a well-organized convective flow across the entire domain. This progressive amplification highlights the direct correlation between input voltage and convective performance, confirming the voltage-dependent control over heat dissipation mechanisms in still air environments. Moreover, the detailed 3D visualizations provide valuable insight into the dynamic behavior of the heat-induced airflow, offering guidance for the design and optimization of thermally functional materials and systems.

#### 3.6.3. Simulation Study with COMSOL Multiphysics of Practical Applications: De-Icing Capability

**Figure 16:**
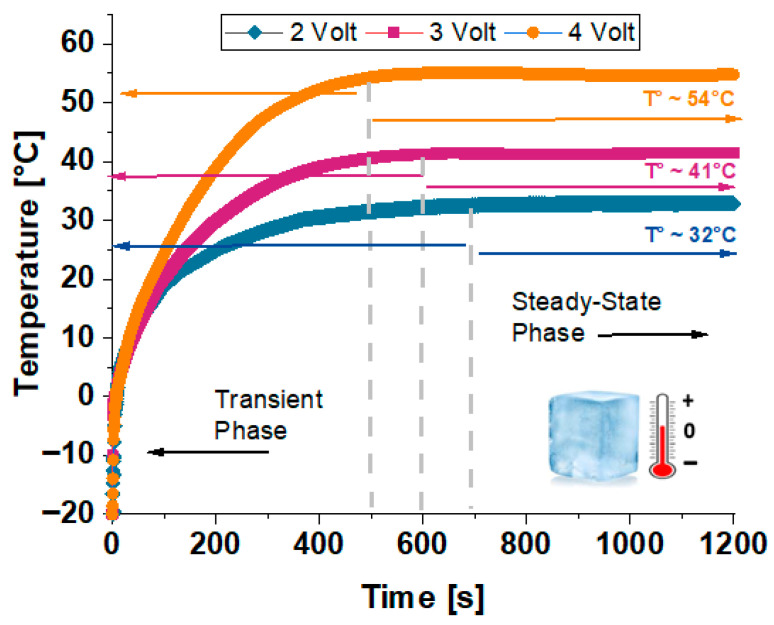
Temperature vs. time curves for the simulated ice block heated by a resistive strip powered at 2 V, 3 V, and 4 V.

In this subsection, the de-icing performance of the most efficient PVDF-based composite formulation (6 wt% of GNP) is investigated through multiphysics simulations. The model replicates, as schematically represented in the previous Figure 3, the melting behavior of an ice cube in direct contact with the composite under applied voltages of 2, 3, and 4 V, highlighting the influence of voltage level on heating rate and thermal response. These simulations offer valuable insights into the practical feasibility of deploying such nanocomposite materials in active de-icing systems for applications requiring efficient thermal management. Thus, Figure 16 shows the temperature evolution of the ice block in contact with the simulated electrically heated strip powered at 2 V, 3 V, and 4 V. All curves exhibit a clear transient phase followed by a steady-state plateau, with both the heating rate and final temperature increasing with voltage. The transient phase shortens and steepens with increasing voltage, consistent with greater Joule heating (Q ∝ V^2^). Final temperatures of ~32 °C, ~41 °C, and ~54 °C were observed for 2 V, 3 V, and 4 V, respectively—values lower than those reached by the bare strip, confirming significant thermal exchange with the ice, including the effect of latent heat. The observed plateaus, therefore, represent the coupled equilibrium between the heating strip and the ice domain, rather than the intrinsic thermal performance of the heating element itself. An important additional observation is that the onset of steady state is reached more rapidly at higher voltages, reflecting the enhanced thermal inertia induced by increased heating power. This implies not only a higher energy input but also a more efficient temporal delivery of thermal energy to the ice, which is critical for applications requiring rapid thawing or deicing. Moreover, the curvature of the heating curves suggests that during the early transient regime, a significant fraction of the thermal input is consumed by the latent heat of fusion, delaying the temperature rise until substantial melting occurs. This effect is more prominent at lower voltages, where the heating rate is insufficient to quickly overcome the enthalpic barrier of the ice phase transition. Consequently, the 2 V curve demonstrates a slower and more prolonged transient phase, highlighting the importance of voltage selection in thermally regulated systems involving phase-change materials. Overall, the results clearly demonstrate that electrical control via voltage tuning offers an effective mechanism to modulate both the kinetics and the thermodynamic endpoint of the ice heating process. This provides useful insights for the design of smart thermal devices, particularly those intended for low-temperature environments or systems requiring controlled thawing profiles.

**Figure 17:**
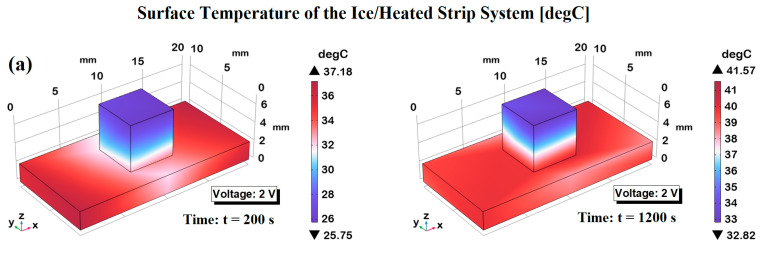

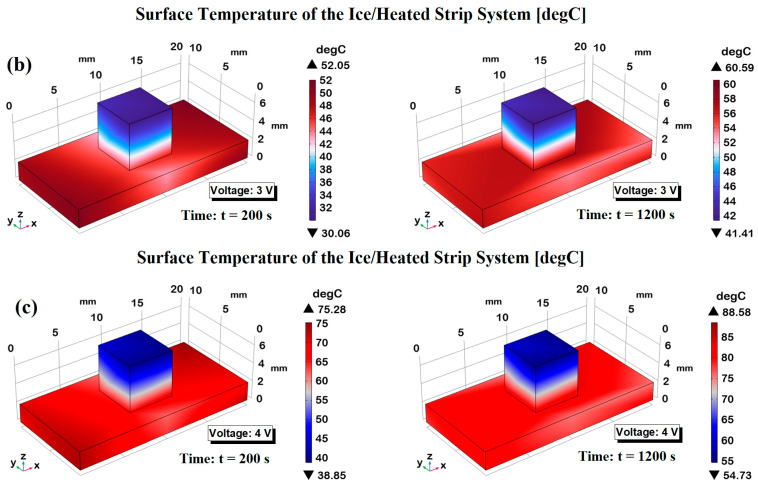
Surface temperature distribution of the ice/heated strip system at three applied voltages: (**a**) 2 V, (**b**) 3 V, and (**c**) 4 V, evaluated at two time points—200 s (**left**), representing the transient heating phase, and 1200 s (**right**), corresponding to steady-state conditions.

Figure 17 illustrates the surface temperature distributions of the system composed of the electrically heated strip in contact with the ice block, under the three considered different voltage levels: 2 V in panel (a), 3 V in panel (b), and 4 V in panel (c). Each condition is captured at two distinct time points: at 200 s (left part), representative of a time instant in the transient phase, and at 1200 s (right part), indicative of the steady-state condition. The simulated system provides insights into the dynamic thermal behavior induced by varying electrical input power.

At 2 V, the heating effect is moderate, with the maximum surface temperature rising from approximately 37 °C at 200 s to just above 41 °C at steady state. At 3 V, the thermal response becomes more pronounced, while the 4 V case exhibits the most significant heating, with temperatures exceeding 88 °C at steady state.

Notably, in all scenarios, even though the ice cube has already melted, the liquid water in contact with the strip remains considerably cooler than the heating element, especially at the top surface of the water volume, which experiences heat losses to the surrounding air and exhibits a slower temperature rise. Overall, the results demonstrate the critical influence of applied voltage on both the spatial and temporal evolution of the temperature field, effectively modulating the rate of ice melting and the subsequent warming of the meltwater. In particular, higher voltages accelerate the transition to steady state and produce a more intense and uniform temperature distribution in the liquid domain, highlighting the system’s potential for tunable thermal control in heat management applications.

**Figure 18:**
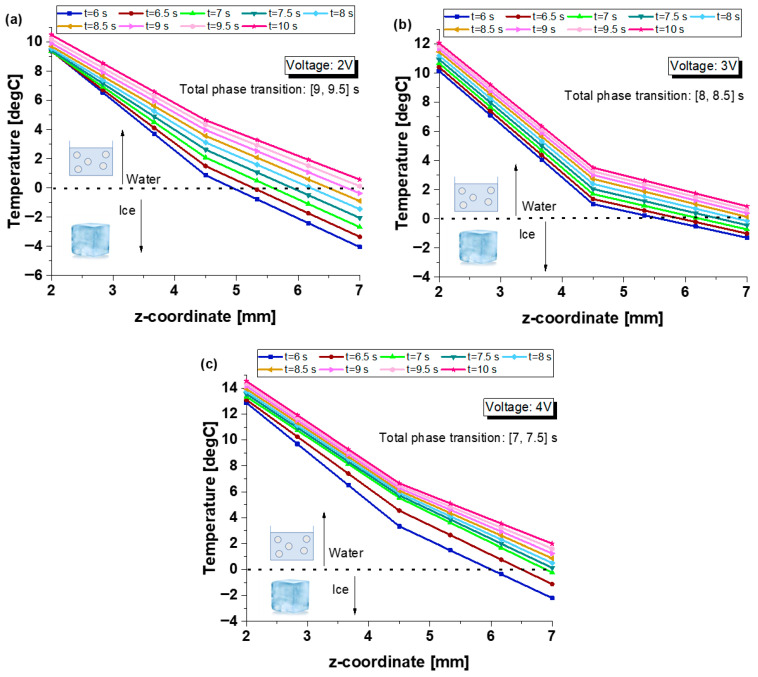
Temperature profiles along the z-axis (symmetry axis) of the ice cube at different time steps (t = 6–10 s) for applied voltages of 2 V (**a**), 3 V (**b**), and 4 V (**c**).

Figure 18 displays the spatial distribution of temperature along the z-axis (symmetry axis) of the ice cube when the resistive strip is subjected to three different applied voltages: 2 V (a), 3 V (b), and 4 V (c). Temperature profiles are reported at successive time steps, from t = 6 s to t = 10 s, thereby capturing the transient heat conduction and the associated melting process of the ice, modeled with the inclusion of latent heat effects. At t = 0 s, the system consists entirely of solid ice, and the liquid water content progressively increases with time.

At 2 V, the temperature gradually increases along the z-axis, with the melting front progressing from the base in contact with the heated strip toward the top. The complete phase transition, i.e., the point at which the entire z-axis reaches temperatures above the melting threshold (0 °C), occurs between 9 and 9.5 s. Increasing the voltage to 3 V accelerates the thermal response: the temperature rises more rapidly along the entire z-axis, and total melting is achieved earlier, between 8 and 8.5 s. At 4 V, the system reaches full melting even faster, between 7 and 7.5 s, indicating a strong dependence of the phase transition rate on the input voltage.

These results clearly demonstrate that higher applied voltages lead to increased heat flux, which in turn accelerates the melting process. The temperature gradients are steeper at earlier times and become more uniform as the ice cube transitions into the liquid phase. Additionally, the inflection point visible in all curves, corresponding to the phase interface, progressively shifts upward over time, confirming the directional nature of melting driven by conductive heat transfer from the heated base. This highlights the system’s responsiveness to controllable electrical input and its potential utility in applications requiring precise thermal management and phase change regulation.

**Figure 19:**
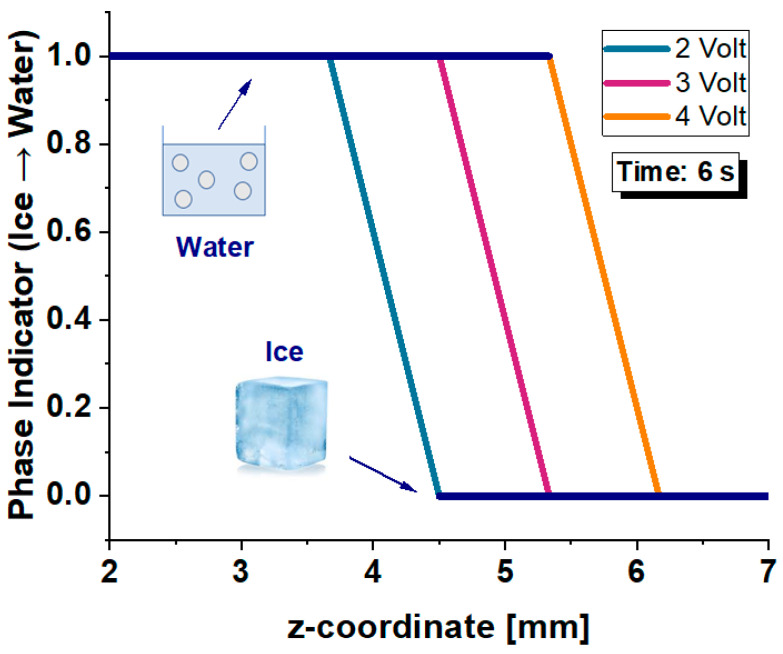
Phase indicator profiles (ice = 0, water = 1) along the z-axis of the ice block at t = 6 s for three applied voltages: 2 V, 3 V, and 4 V.

Figure 19 illustrates the spatial progression of the solid-to-liquid phase transition along the z-axis of the ice cube at a fixed time of 6 s, under the three different applied voltages: 2 V, 3 V, and 4 V. The phase indicator, which ranges from 0 (solid ice) to 1 (fully liquid water), clearly delineates the extent of melting induced by each voltage level. At this intermediate stage of the phase change, a distinct shift in the melting front toward higher z-coordinates is observed with increasing voltage. Specifically, the 4 V case exhibits the most advanced phase boundary, indicating a larger portion of the ice volume has already undergone complete melting.

These results confirm that higher voltages significantly enhance the rate of phase change, driven by intensified Joule heating and consequent heat conduction from the bottom heated layer. The steep gradients of the phase indicator curves suggest a sharp and well-defined melting front, consistent with conductive-dominated heat transfer. This spatial shift in the solid–liquid interface underscores the controllability of the melting dynamics through electrical input and demonstrates the system’s potential for programmable thermal regulation in phase-change-based applications such as thermal switches, microfluidic control, or energy storage systems.

## 4. Conclusions

This work presents a robust, multi-scale investigation of the electrothermal properties of PVDF-based nanocomposites reinforced with MWCNTs, GNPs, and their hybrids, revealing clear correlations between filler architecture, dispersion, and functional performance. Through a combination of statistically designed experiments and analytical modeling via Response Surface Methodology (RSM), it was possible to successfully establish a predictive relationship between filler content and both electrical and thermal conductivity, thus offering a design-oriented tool that goes beyond empirical observations typically found in the literature. Among all configurations, the nanocomposite containing 6 wt% GNPs exhibited exceptional performance, achieving the highest conductivity and most efficient, homogeneous Joule heating under moderate voltage conditions. This formulation not only surpassed others in thermal response rate and stability but also proved to be highly reproducible and compatible with scalable additive manufacturing processes. Multiphysics simulations, validated against experimental and theoretical benchmarks, confirmed the capability of the optimized material to achieve rapid de-icing through localized heating, demonstrating its applicability in demanding thermal management scenarios. These results position our material as a viable candidate for integration into smart systems, including aerospace components, wearable electronics, and energy-efficient de-icing layers. By bridging experimental insight, analytical rigor, and numerical validation, this study contributes a novel and generalizable methodology for the rational design of advanced electrothermal composites, establishing a solid foundation for their deployment in next-generation functional materials.

## Figures and Tables

**Table 1:** Characteristics of the carbon nanofillers, GNPs, and MWCNTs.

Filler	GNP	MWCNT
Trade Name	SE1233	NC7000
Purity, C wt.%	>97	90
Average size, D_50_, μm	35–50	-
Thickness, nm	<10	-
Length, μm	-	1.5
Outer diameter, nm		9.5
Aspect ratio	3500–5000	150
Shape; Surface Area (SSA) m^2^/g	2D; 400–600	1D; 250–300
Volume Resistivity, Ω·cm	-	10^−4^

**Table 2:** Nanocomposites were produced for this study.

Samples	PVDF wt.%	GNP wt.%	MWCNT wt.%
6GNP/PVDF	94.0	6.0	0
4.5GNP/1.5MWCNT	94.0	4.5	1.5
3GNP/3MWCNT	94.0	3.0	3.0
1.5GNP/4.5MWCNT	94.0	1.5	4.5
6MWCNT/PVDF	94.0	0	6.0

**Table 3:** Prescribed initial and boundary conditions necessary for the solution of the thermal energy equation.

Initial (I.C.) and Boundary (B.C.) Conditions		Equations	Validity
I.C.	t = 0	T = Room Temperature (T_0_)	∀x,∀y,∀z
B.C.	Topside and underside z = 0 z = 2	$-\lambda \frac{\partial T}{\partial z}=h\cdot \left(T-T_{\infty }\right)$	(∀x,∀y,t>0)
B.C.	Lateral Surfaces $\begin{array}{c} y=0\\ \;y=20 \end{array}$	$-\lambda \frac{\partial T}{\partial y}=h\cdot \left(T-T_{\infty }\right)$	(∀x,∀z,t>0)
B.C.	Backside and frontside $\begin{array}{c} x=0\\ \;x=10 \end{array}$	$-\lambda \frac{\partial T}{\partial x}=h\cdot \left(T-T_{\infty }\right)$	(∀y,∀z,t>0)

**Table 4:** Physical properties of ice and water.

Physical Property.	Ice	Water
Density [kg/m^3^]	918	997
Heat capacity at constant pressure [J·kg^−1^·K^−1^]	2052	4179
Thermal conductivity [Wm^−1^·K^−1^]	2.31	0.613

**Table 5:** Joule heating characteristics.

Physical Property Sample	Voltage [V]	Max. Temp. [°C]	Max. Curr. [A]	Power [W]	Gen. Heat [J]	Heat Rate [°C/s]	Heat Efficiency [%]
6GNP/PVDF	2	41.2	1.12 × 10^−1^	2.23 × 10^−1^	1.12 × 10^2^	1.25 × 10^−1^	3.93 × 10
	3	60.3	1.67 × 10^−1^	5.02 × 10^−1^	2.51 × 10^2^	2.19 × 10^−1^	5.85 × 10
	4	88.2	2.23 × 10^−1^	8.93 × 10^−1^	4.46 × 10^2^	4.38 × 10^−1^	7.17 × 10
4.5GNP/1.5MWCNT	2	38.23	9.78 × 10^−2^	1.96 × 10^−1^	9.78 × 10	8.00 × 10^−2^	3.46 × 10
	3	53.1	1.47 × 10^−1^	4.40 × 10^−1^	2.20 × 10^2^	1.71 × 10^−1^	5.29 × 10
	4	72.3	1.96 × 10^−1^	7.82 × 10^−1^	3.91 × 10^2^	3.05 × 10^−1^	6.54 × 10
3GNP/3MWCNT	2	36.4	9.62 × 10^−2^	1.92 × 10^−1^	9.62 × 10^1^	1.14 × 10^−1^	3.13 × 10
	3	52.0	1.44 × 10^−1^	4.33 × 10^−1^	2.16 × 10^2^	1.91 × 10^−1^	5.19 × 10
	4	69.5	1.92 × 10^−1^	7.70 × 10^−1^	3.85 × 10^2^	3.24 × 10^−1^	6.43 × 10
1.5GNP/4.5MWCNT	2	39.1	1.02 × 10^−1^	2.04 × 10^−1^	1.02 × 10^2^	1.14 × 10^−1^	3.61 × 10
	3	57.6	1.53 × 10^−1^	4.60 × 10^−1^	2.30 × 10^2^	2.21 × 10^−1^	5.66 × 10
	4	82.6	2.04 × 10^−1^	8.18 × 10^−1^	4.09 × 10^2^	4.36 × 10^−1^	6.98 × 10
6MWCNT/PVDF	2	39.5	1.04 × 10^−1^	2.08 × 10^−1^	1.04 × 10^2^	1.04 × 10^−1^	3.67 × 10
	3	56.6	1.56 × 10^−1^	4.69 × 10^−1^	2.34 × 10^2^	2.00 × 10^−1^	5.58 × 10
	4	76.0	2.08 × 10^−1^	8.34 × 10^−1^	4.17 × 10^2^	4.09 × 10^−1^	6.74 × 10

**Table 6:** Coefficients for the full quadratic response of the electrical and thermal conductivity as determined by RSM.

Property	β_0_	β_1_	β_2_	β_11_	β_22_	β_12_
Electrical Conductivity (σ)	0.19801	0.58936	0.59873	2.1688	1.3674	1.4236
Thermal Conductivity (λ)	0.00086245	0.0023666	0.0028081	0.0092595	0.0049403	0.007589

**Table 7:** Comparison between simulated and experimental results, including relative error percentage (% Change), for the plateau temperature values (T_p_) obtained at different voltage levels.

Voltage [V]	Experimental T_P_ [°C]	Numerical T_P_ [°C]	% Change
2	41.2	41.57	0.898
3	60.3	60.59	0.481
4	88.2	88.58	0.431

## Data Availability

The original contributions presented in this study are included in the article. Further inquiries can be directed to the corresponding authors.
